# Sperm Protein Antigen 17 Expression Correlates With Lymph Node Metastasis and Worse Overall Survival in Patients With Breast Cancer

**DOI:** 10.3389/fonc.2019.00710

**Published:** 2019-07-31

**Authors:** Yu-ting Zhou, Juan-juan Qiu, Yao Wang, Peng-cheng Liu, Qing Lv, Zheng-gui Du

**Affiliations:** West China Hospital, Sichuan University, Chengdu, China

**Keywords:** sperm protein antigen 17, cancer-testis antigens, migration, invasion, breast cancer

## Abstract

**Purpose:** The expression and role of sperm protein antigen 17 (SPA17), which has been confirmed to be immunogenic, in breast cancer remain unclear. We examined the expression of SPA17 in breast cancer and assessed its effect on patient prognosis and its function in breast cancer development.

**Methods:** SPA17 expression was evaluated by immunohistochemistry and Q-RT-PCR in 120 breast tissue samples. Correlation of SPA17 expression with the patients' clinicopathological parameters and overall survival was assessed. The function of SPA17 was also explored.

**Results:** By reviewing Gene Expression Omnibus datasets, we found that SPA17 expression in ductal breast carcinoma in situ (log2[fold change] = 1.14, *p*-value = 0.004) and invasive ductal breast cancer (log2[fold change] = 1.03, *p*-value = 0.016) tissues was 2.20 and 2.05 times higher, respectively, than that in normal breast tissues. Our result also showed that 27% (27/100) of breast cancer samples expressed SPA17 but none of the normal breast (0/20) samples did. Lymph node metastasis (*p* < 0.001) and molecular subtyping (*p* = 0.002) were independent factors associated with SPA17 expression. Most importantly, SPA17 expression resulted in poor prognosis. In addition, cell function assay validated that SPA17 increased the migration (*p* < 0.001) and invasion (*p* = 0.007) of breast cancer cells, but not affected the proliferation of breast cancer cells.

**Conclusion:** Our results demonstrated the vital role of SPA17 in the development and metastasis of breast cancer and that SPA17 may be a new therapeutic target in improving breast cancer prognosis.

## Introduction

The morbidity of breast cancer has been significantly increasing worldwide during the last 30 years. And for now, but breast cancer is the most diagnosed in both American and Chinese women (1). Not only that, but breast cancer has also been the second leading cause of death due to cancer in America, and the sixth in China (2, 3). Therefore, breast cancer carries a huge burden for both patients and society. The existing treatment regimens, including surgery, chemotherapy, radiotherapy, endocrine therapy, and/or targeted therapy, can hardly reduce the mortality of breast cancer patients any further (4, 5). Hence, more and more studies are concentrating on immunotherapy and the discovery of novel therapeutic targets (6).

With the rapid development of immunotherapy in the past two decades, many tumor-associated antigens were discovered. Cancer-testis antigens (CTAs) are a special group of tumor-associated antigens that are only expressed in the human testis germ cells and a number tumors, such as melanoma (7), non-small cell lung cancer (8), ovarian cancer (9, 10), endometrial carcinoma (11), but not in normal tissue (12). Sperm protein antigen 17 (SPA17) is a member of CTAs expressed in many of cancers mentioned above (13–16), especially in ovarian cancer (17). Recently, Song et al. generated SPA17-specific, HLA class I-restricted, cytotoxic T lymphocytes capable of efficiently killing breast cancer cells. And a previous study has also confirmed SPA17 antibody can effectively inhibit the growth of human ovarian cancer cells SKOV-3 (18). Moreover, Liu et al. found umbilical cord blood-derived dendritic cells modulated for SPA17 expression induced antigen-specific anti-tumor immunity against SPA17 positive non-small cell lung cancer (19), but the role of SPA17 in cancer development, especially in breast cancer development, is still not clear.

Therefore, in this study, we first use the Gene Expression Omnibus and Oncomine public databases to explore the differences of SPA17 expression in breast cancer tissues and normal breast tissues, and further validate and analyze whether SPA17 expression is associated with the stage and prognosis of breast cancer. If so, we finally focus on the mechanisms by which SPA17 affects the prognosis of breast cancer through cytological studies.

## Methods

### Microarray Data From GEO and Oncomine Datasets

We obtained the gene expression profile of GSE21422 from Gene Expression Omnibus (http://www.ncbi.nlm.nih.gov/geo/), a free and publicly available database. The GSE21422 dataset was based on the GPL570 platform ([HG-U133_Plus_2] Affymetrix Human Genome U133 Plus 2.0 Array). We use the “limma” R package to screen for SPA17 between breast cancer samples and normal breast tissue samples. False discovery rate (FDR) < 0.05 and |log2fold change (FC)| > 1 were chosen as the cut-off criteria.

### Clinical Specimens and Collection of Clinicopathological Parameters as Well as Follow-Up Information

The 100 samples of primary breast cancer and 20 normal breast specimens (from non-tumor bearing tissue) analyzed in our study were obtained from patients who had undergone surgical treatment at the Department of Breast Surgery, West China Hospital, Sichuan University between June and August 2011. We excluded patients who had received preoperative adjuvant chemotherapy and radiotherapy, had recurrence and those without a complete pathology diagnostic report. Clinicopathological parameters of the patients with breast cancer were collected by reviewing their medical records. Annual follow-up was performed for each patient after surgery to ensure reliable prognostic information was obtained. Informed consent was obtained from all individuals included in the study. The ethical approval was obtained from the ethics committee of the Sichuan University.

### Cell Culture

The human breast cancer cell line MCF-7 and MDA-MB-231 were purchased from American Type Culture Collection. The cells were cultured in high-glucose Dulbecco's modified Eagle medium (DMEM, Gibco) supplemented with 10% fetal bovine serum (FBS, Gibco) in a humidified atmosphere of 5% CO2 at 37°C.

### Establishment of MCF-7 Breast Cancer Cells With Overexpression of SPA17 and MDA-MB-231 Breast Cancer Cells With Silencing of SPA17

To explore the role of SPA17 in breast cancer invasion and metastasis, a SPA17 and enhanced green fluorescent protein (EGFP) co-expression plasmid SPA17-3Flag-IRES-EGFP (He Yuan Biology, Shanghai, China) was constructed and transfected into the MCF-7 cells in which SPA17 is expressed at low levels. *In vitro* DNA transfection was performed using Lipofectamine 3000 (Invitrogen). MCF-7 cells were incubated in RPMI opti-MEM (Gibco) at 37°C in an atmosphere of 5% CO2 for 12 h, and the media was then replaced with DMEM containing 10% FBS. The cells were infected with plasmids containing either SPA17 cDNA or EGFP cDNA, denoted as SPA17-MCF-7 and 3Flag-MCF-7, respectively. For siRNAs transfection, RPMI opti-MEM (Gibco), Lipofectamine 3000 (Invitrogen) reagent and siRNAs targeting human SPA17, or control siRNA (non-specific sequences) (He Yuan Biology, Shanghai, China) mixtures were prepared, respectively. According to the instructions of the manufacture, the mixtures was added in each group. The gene and protein expression of SPA17 in the above cells were confirmed using quantitative polymerase chain reaction (Q-PCR) and western blot.

### Quantitative Real-Time PCR, Western-Blot and Immunohistochemistry

Quantitative real-time PCR, western-blot and immunohistochemistry were conducted as described previously (16, 17, 20). cDNA was amplified using the following primers: SPA17: Forward: ATTCTCCAACACCCACTA Reverse: GGTCTTCTACCTTACTCCC β-actin: Forward: ACTTAGTTGCGTTACACCCTT Reverse: GTCACCTTCACCGTTCCA (Sangon Biotech, Shanghai, China).

The proteins in MCF-7, SPA17-MCF-7, 3Flag-MCF-7, MDA-MB-231, MDA-MB-231-SPA17-siRNA#control [5′-GCGUAACGCGGGAAUUUACUU-3′ (sense) and 5′-GUAAAUUCCCGCGUUACGCUU-3′ (antisense)], MDA-MB-231-SPA17-siRNA#1 [5′-AAAAAAAAAAAAACUACGGCG-3′ (sense) and 5′-CCGUAGUUUUUUUUUUUUUUU-3′ (antisense)], MDA-MB-231-SPA17-siRNA#2 [5′-GCAUCACAGUGCUAAGCAAUA-3′ (sense) and 5′-UUGCUUAGCACUGUGAUGCUG-3′ (antisense)], MDA-MB-231-SPA17-siRNA#3 [5′-UAUAGAAGCGGUCUUCUACCU-3′ (sense) and 5′-GUAGAAGACCGCUUCUAUAAC-3′ (antisense)], and cells were extracted with the Membrane and Cytosol Protein Extraction Kit (Beyotime, Shanghai, China). Protein concentrations were determined with an Enhanced BCA Protein Assay Kit (Beyotime, Shanghai, China). SPA17 antibodies, β-actin antibodies, and horseradish peroxidase-conjugated anti-mouse secondary antibodies were purchased from Novus Biologicals (H00053340-B01P, Cambridge, UK), Santa Cruz Biotechnology (sc-47778, Santa Cruz, CA), and ZSGB-BIO(AT-09, Beijing, China), respectively.

The immunohistochemical reaction was observed using light microscope (ZEISS, Germany) at 20× and 40× objective magnifications, and was semi-quantitatively graded into four classes by two pathology experts based on the intensity of SPA17 staining in breast cancer cells: negative = no immunopositive cells (x = 0); + = low frequency (x ≤ 25%); ++ = moderate frequency (25% <x ≤ 75%); +++ = high frequency (75% <x ≤ 100%).

### Cell Proliferation, Migration and Invasion Assay

For proliferation assays, cell suspensions were plated in 96-well plate (3,000 cells/well) in quadruplicate and evaluated following a period of incubation (24, 48, 72, and 96 h). Cells were incubated with medium containing 10 μl of 3-(4,5-dimethylthiazol-2-yl)−2,5-diphenyltetrazolium bromide (MTT, 5 mg/ml; Sigma-Aldrich, Germany, Cat No. M5655) for 3 h at 37°C, medium was removed and formazan crystals dissolved in 200 μL DMSO. Subsequently, cell viability was determined at the wavelength of 450 nm using a spectrophotometer (BioTek Instruments, Inc., Winooski, VT, USA).Data were derived from at least three independent experiments.

Cells (1 × 10^6^/ well) were seeded into a 6-well plate and incubated for 24 h. When the cells almost reached confluence, they were wounded with a plastic tip and cultured with DMEM supplemented with 10% FBS at 37°C in 5% CO2 for 48 h. The gaps and cells were imaged at 0 h, 24 h and 48 h. Wound healing was measured as described (21). Ten random micrographs per well were obtained and migration area was quantified using Image J. software (https://imagej.nih.gov/ij/). Wound-closure measurements were normalized to the maximum “scratch” area. For the Transwell assay, cells were resuspended in serum-free DMEM at 5 × 10^5^ cells/mL, and a 150-mL cell suspension was seeded into the upper chamber (Corning Costar, Tewksbury, MA, USA), to which 60 μL of Matrigel was added at a concentration of 0.8 g/L. The chamber was placed in 24-well plates and DMEM containing 10% FBS was added to the bottom wells. After incubation for 48 h at 37°C in 5% CO2, non-invaded cells and Matrigel were removed from the top side of the inserts with a cotton swab. The invaded cells on the lower chamber were fixed with 100% methanol, stained with crystal violet for 30 mins, and photographed. Six randomly chosen fields were analyzed for each well.

### Statistical Analysis

Descriptive statistics included means, ranges and standard deviations. Categorical data were presented as percentages. Differences between proportions were compared using the Chi-square or Fisher exact tests. Continuous variables were compared using the unpaired Student *t*-test. Multivariate analysis was performed to identify independent determinants for SPA17 expression. ORs with 95% confidence interval (CIs) for overall survival (OS) and disease-free survival (DFS) were generated with the Cox regression analysis. Kaplan–Meier regression was conducted to analyze whether SPA17 expression affected prognosis in patients with breast cancer. All statistical evaluations were performed using SPSS for Windows (SPSS 25.0, Chicago, IL). Results with a *P* <0.05 were considered statistically significant.

## Results

### Expression of SPA17 Associated With the Occurrence of Breast Cancer

**Figure 1:**
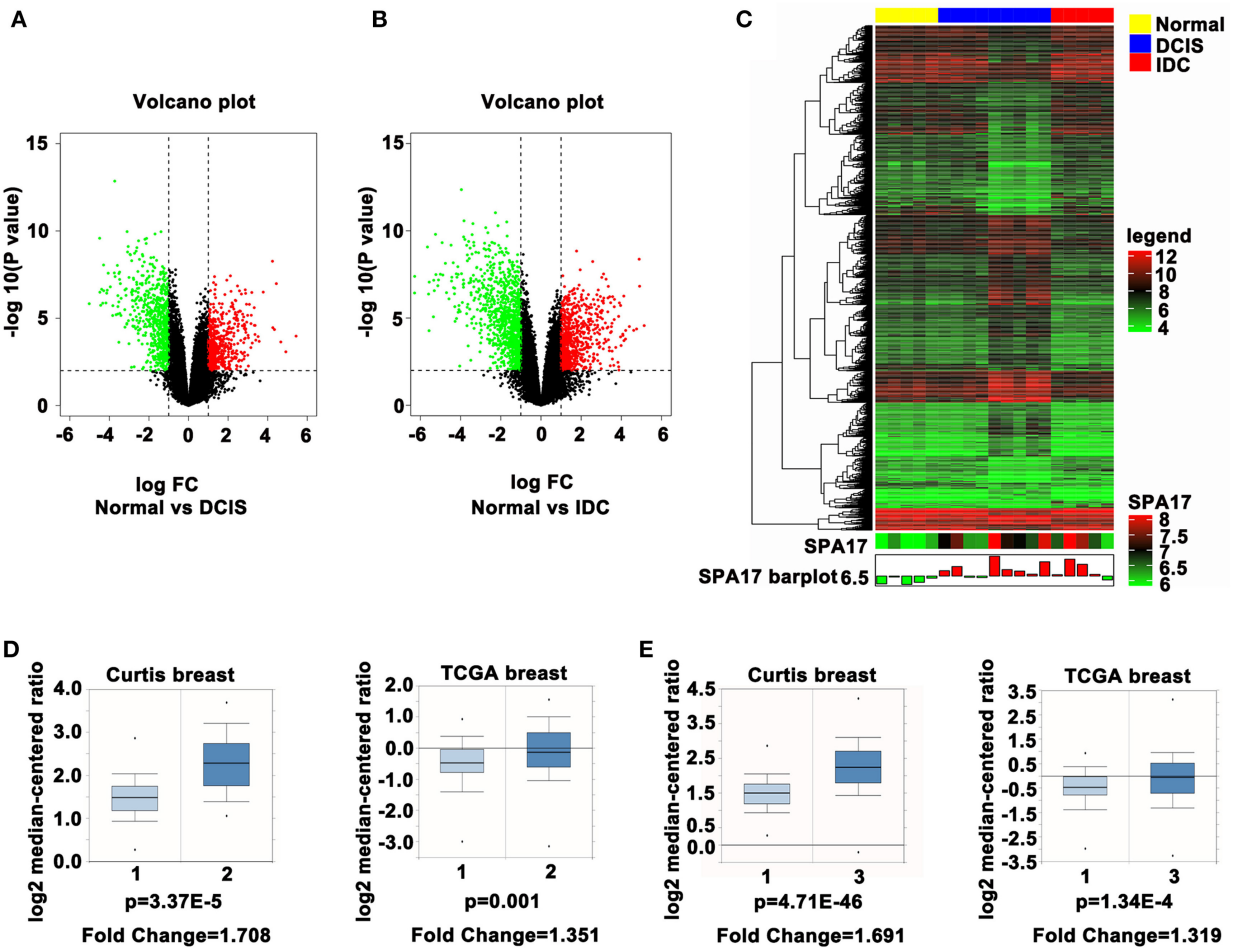
**(A)** The volcano plot of SPA17 expression in DCIS vs. normal breast tissues and **(B)** IDC vs. normal breast tissues (DCIS = ductal carcinoma *in situ*, IDC = invasive ductal carcinoma). **(C)** Heatmap plot of SPA17 expression in breast cancer and normal breast tissues. **(D)** SPA17 expression in invasive breast carcinoma (2) vs. normal breast tissue (1) in Curtis (*p* = 3.37E-5, fold change = 1.708) and in TCGA Breast Database (*p* = 0.001, fold change = 1.351). **(E)** SPA17 expression in invasive ductal breast carcinoma (3) vs. normal breast tissue (1) in Curtis (*p* = 4.71E-46, fold change = 1.691) and in TCGA Breast Database (*p* = 1.34E-4, fold change = 1.319).

In order to dynamically study the difference in SPA17 expression between breast cancer tissues and normal breast tissues, we downloaded the GSE21422 dataset, which contains 9 ductal breast carcinoma in situ (DCIS) samples, 5 invasive ductal breast cancer (IDC) samples, and 5 normal breast tissue samples. First, we used the “limma” R package to perform differential gene analysis and screen for differential genes among the three groups and constructed volcano plots and heatmap (Figures 1A–C). The expression of SPA17 in DCIS (log_2_[fold change (FC)] = 1.14, p-value = 0.004) and IDC (log_2_FC = 1.03, p-value = 0.016) samples was found to be 2.20 and 2.05 times higher, respectively, than that in normal breast tissues. There was no significant difference in the expression of SPA17 between the DCIS and IDC groups (*p* = 0.771) (Figure 1C). Moreover, we reviewed the Oncomine database and found that in the Curtis and The Cancer Genome Atlas datasets, the expression of SPA17 in invasive breast carcinoma was higher than that in normal breast, and the difference was statistically significant (Figure 1D). A similar phenomenon was observed in IDC (Figure 1E).

**Figure 2:**
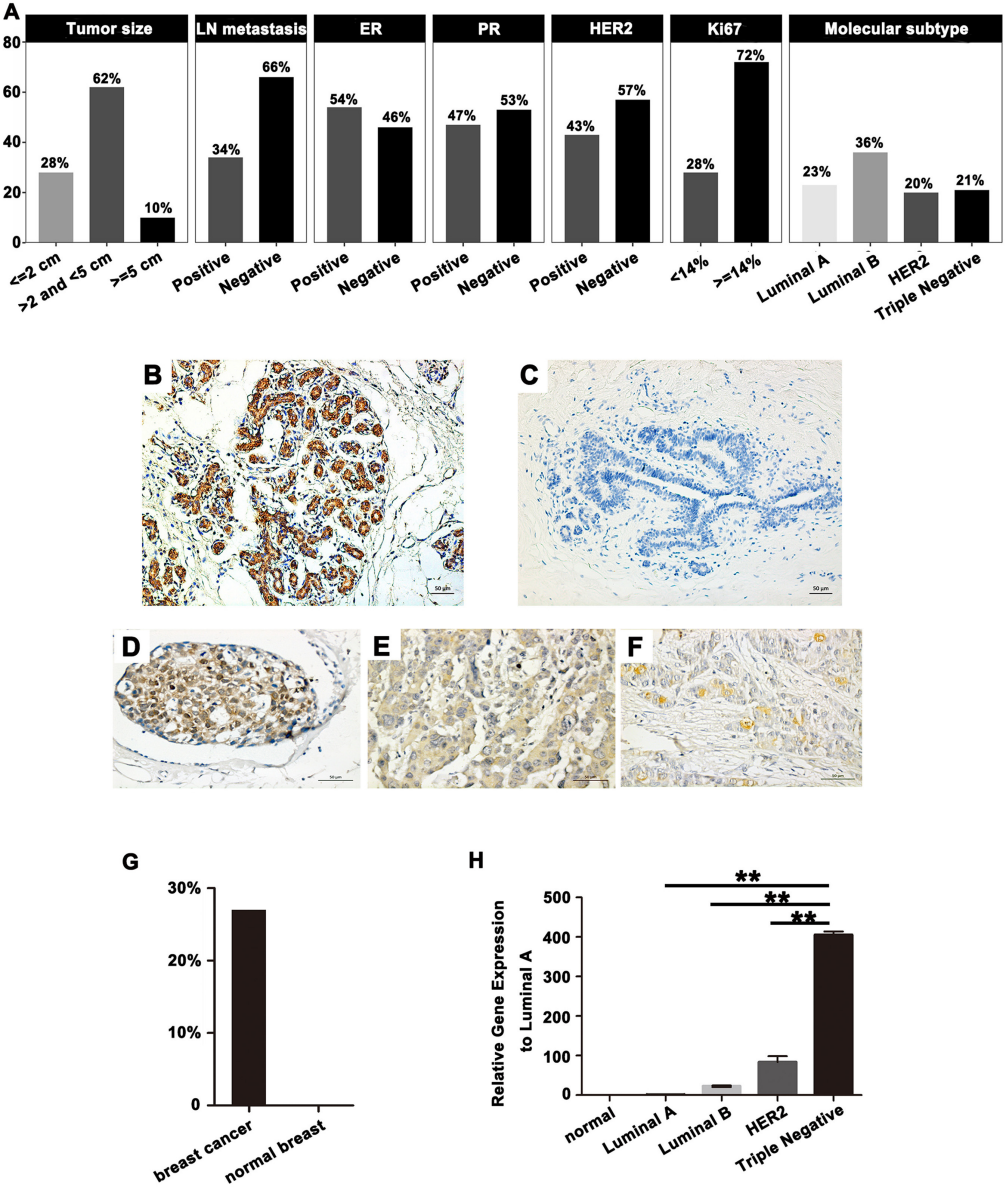
**(A)** Basic clinicopathological features in 100 breast cancer patients. SPA17 protein expression in **(B)** normal human testes, **(C)** normal breast tissue, and **(D)** low frequency (x ≤ 25%); **(E)** moderate frequency (25% <x ≤ 75%); **(F)** high frequency (75% <x ≤ 100%) breast cancer specimens. Human testis was the positive control. **(G)** SPA17 protein expression rate in breast cancer and normal breast tissue. **(H)** SPA17 mRNA expression in breast cancer tissues and normal breast tissues. ^*^p < 0.05, ^**^p < 0.01.

The expression of SPA17 mRNA and protein were further determined in 100 breast cancer specimens and 20 normal breast tissues by quantitative real-time PCR and immunohistochemistry. All the patients reviewed in this study were female with a median age of 50.8 years (range: 28 to 89 years), and were diagnosed with invasive breast cancer. More than sixty percent patients had tumors larger than 2 cm. The number of patients with luminal breast cancer was slightly higher than non-luminal breast cancer. The median follow-up time was 58 months (range: 6–72). Details are shown in Figure 2A.

The results showed SPA17 protein was expressed in 27% (27/100) of breast cancer specimens and in none of the normal breast samples (Figures 2B–G). The expression of SPA17 mRNA in breast cancer specimens was similar to that of SPA17 protein, but not expressed in normal breast samples (Figure 2H). All of these results provided strong support for us to further explore the function of SPA17 in breast cancer.

### Expression of SPA17 Associated With the Development of Breast Cancer

**Figure 3:**
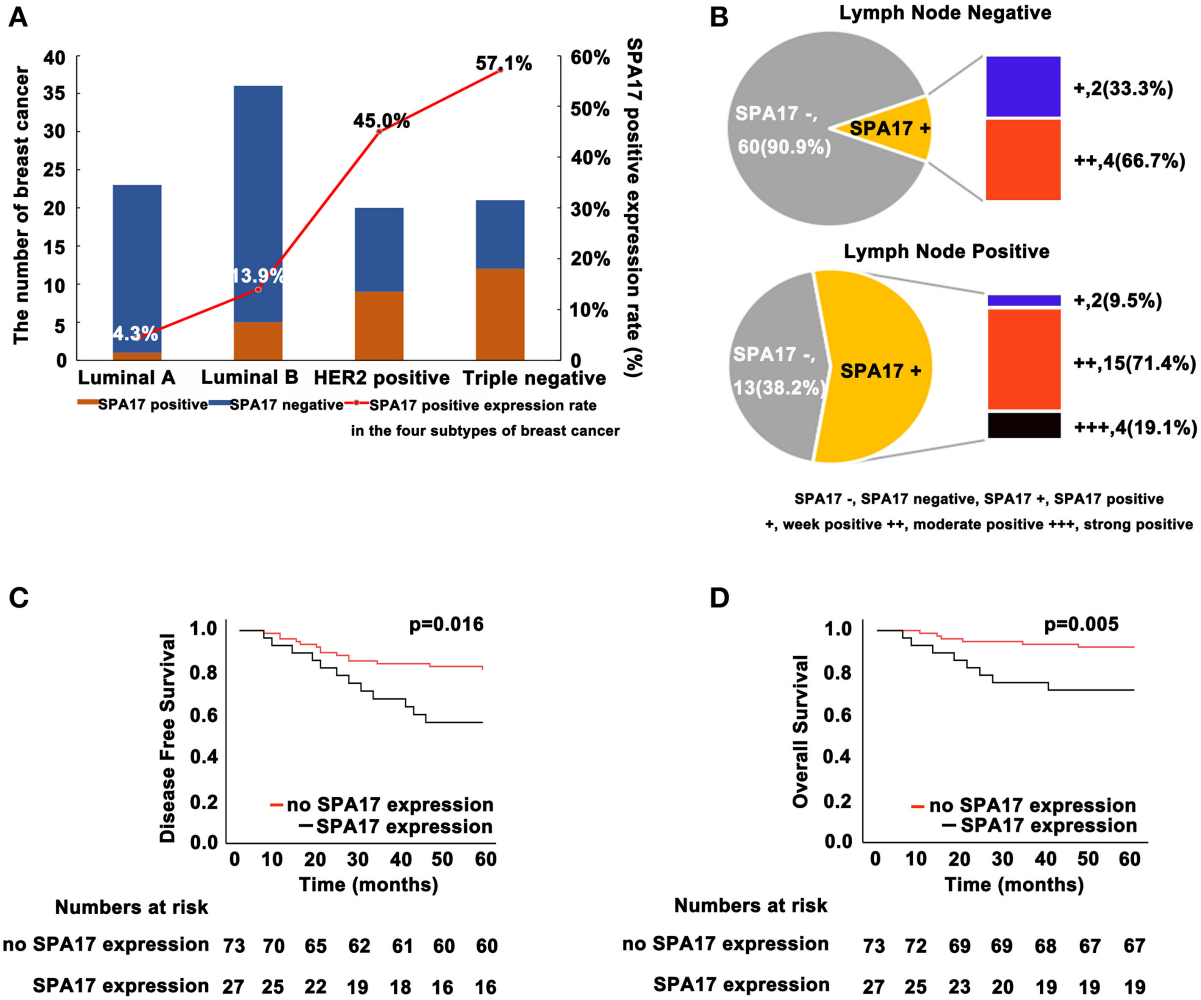
**(A)** SPA17 protein expression status in four molecular subtypes of breast cancer. SPA17 protein expression was positive in the majority of non-luminal breast cancer tissues. **(B)** SPA17 protein expression in lymph node negative and lymph node positive breast cancer patients. **(C)** SPA17 positive expression is associated with lower disease-free survival and **(D)** overall survival among breast cancer patients.

Our analyses further revealed that the expression of SPA17 was associated with lymph node metastasis (*p* < 0.001), ER status (*p* < 0.001), PR status (*p* = 0.003), molecular subtyping (*p* < 0.001), Endocrine-therapy (*p* < 0.001) and Radiotherapy (*p* < 0.001) (Table 1). About 77.8% (21/27) of patients with SPA17 expression had lymph node metastasis. SPA17 mRNA and protein expression were positive in the majority of non-luminal breast cancer samples (Figures 2H, 3A). Further analysis revealed that 61.8% (21/34) of patients with lymph node metastasis had positive SPA17 expression (Figure 3B). These results indicated that SPA17 might relate to the metastasis of breast cancer cells, thus affecting the prognosis of breast cancer patients. As a potential prognostic and theraputic target, we did the Cox regression univariate analysis, the results showed SPA17, lymph node metastasis, tumor size, ER status, PR status, molecular subtyping, endocrine-therapy and radiotherapy were associated with overall survival. While SPA17, lymph node metastasis, tumor size, PR status, Ki-67 status, molecular subtyping, endocrine-therapy and radiotherapy were associated with disease-free survival (Tables 2, 3). All factors with *p* < 0.1 were included in the Cox regression multivariate analysis. Further multivariate analysis showed SPA17 and tumor size were independent factors associated with overall survival. SPA17, tumor size and endocrine-therapy were independent factors related to disease-free survival (Tables 2, 3).

**Table 1:** Association between Sp17 expression and clinicopathological features—univariate analysis.

**Clinicopathological features**	**Sp17**		**t**	**X^**2**^**	***p***
	**+**	**−**			
Ages (years)	49.70 ± 7.8	51.3 ± 10.8	−0.683		0.496
Tumor size (cm)				3.572	0.168
≤ 2	4	24			
>2 and <5	19	43			
≥5	4	6			
Lymph node metastasis				31.588	<0.001
Yes	21	13			
No	6	60			
ER status				16.056	<0.001
Positive	6	49			
Negative	21	24			
PR status				9.116	0.003
Positive	6	41			
Negative	21	32			
HER2 status				1.182	0.277
Positive	14	29			
Negative	13	44			
Ki-67 status				3.190	0.074
<14%	4	24			
>14%	23	49			
Molecular subtyping				22.096	<0.001
Luminal A	1	22			
Luminal B	5	31			
HER2-	9	11			
positive					
Triple-	12	9			
negative					
Chemotherapy				0.609	0.435
Yes	22	54			
No	5	19			
Endocrine-therapy					
Yes	5	43		13.749	<0.001
No	17	20			
Radiotherapy				16.295	<0.001
Yes	17	15			
No	10	58			

**Table 2:** Association between clinicopathological features and overall survival—Cox regression analysis.

**Variables**	**Univariate analysis**	***p*-value**	**Multivariate analysis**	
	**HR (95% CI)**		**HR (95% CI)**	***p*-value**
Age (years)	0.971(0.919–1.025)	0.280		
SPA17	4.108(1.424–11.851)	0.009	3.902(1.216–12.525)	0.022
Lymph node metastasis	3.936(1.318–11.754)	0.014		
Tumor size(cm)		0.009		0.019
≤2	1		1	
>2 and <5	1.660(0.345–7.990)	0.527	2.021(0.239–17.093)	0.518
≥5	8.023(1.554–41.417)	0.013	9.352(1.062–82.359)	0.044
ER status	0.285(0.089–0.910)	0.034		
PR status	0.275(0.077–0.987)	0.048		
HER2 status	1.349(0.473–3.845)	0.576		
Ki-67 status	35.437(0.287–4370.796)	0.146		
Molecular subtyping		0.096		
Luminal A	1			
Luminal B	1.939(0.202–18.645)	0.566		
HER-2	7.835(0.943–65.140)	0.057		
Triple-negative	5.333(0.596–47.731)	0.134		
Chemotherapy	4.469(0.585–34.168)	0.149		
Endocrine-therapy	7.312(1.600–33.405)	0.010		
Radiotherapy	4.509(1.510−13.468)	0.007		

**Table 3:** Association between clinicopathological features and disease-free survival—Cox regression analysis.

**Variables**	**Univariate analysis**	***p*-value**	**Multivariate analysis**	
	**HR (95% CI)**		**HR (95% CI)**	***p*-value**
Age (years)	0.985(0.948–1.023)	0.433		
SPA17	2.713(1.252–5.877)	0.011	2.527(1.092–5.845)	0.030
Lymph node metastasis	2.164(1.002–4.673)	0.050		
Tumor size(cm)		0.004		0.033
≤2	1		1	
>2 and <5	4.429(1.023–19.175)	0.047	5.410(0.710–41.231)	0.103
≥5	12.343(2.557–59.581)	0.002	12.464(1.513–102.701)	0.019
ER status	0.540(0.248–1.175)	0.120		
PR status	0.455(0.198–1.047)	0.064		
HER2 status	1.427(0.661–3.078)	0.365		
Ki-67 status	3.348(1.005–11.154)	0.049		
Molecular subtyping		0.098		
Luminal A	1			
Luminal B	1.590(0.411–6.150)	0.501		
HER-2	3.579(0.949–13.498)	0.060		
Triple-negative	3.763(0.997–14.204)	0.051		
Chemotherapy	1.155(0.464–2.878)	0.756		
Endocrine-therapy	4.433(1.745–11.264)	0.002	2.624(1.013–6.798)	0.047
Radiotherapy	2.092(0.966–4.530)	0.061		

Kaplan-Meier analysis showed that the differences in DFS (*p* = 0.016, Figure 3C) and OS (*p* = 0.005, Figure 3D) in patients with and without SPA17 expression were statistically significant. Since SPA17 expression was more frequently observed in samples from patients with lymph node metastasis of breast cancer and the triple negative and HER2 positive breast cancer specimens, we supposed that SPA17 expression might affect the prognosis of breast cancer patients.

### SPA17 Increased the Migration and Proliferation of Breast Cancer Cells

**Figure 4:**
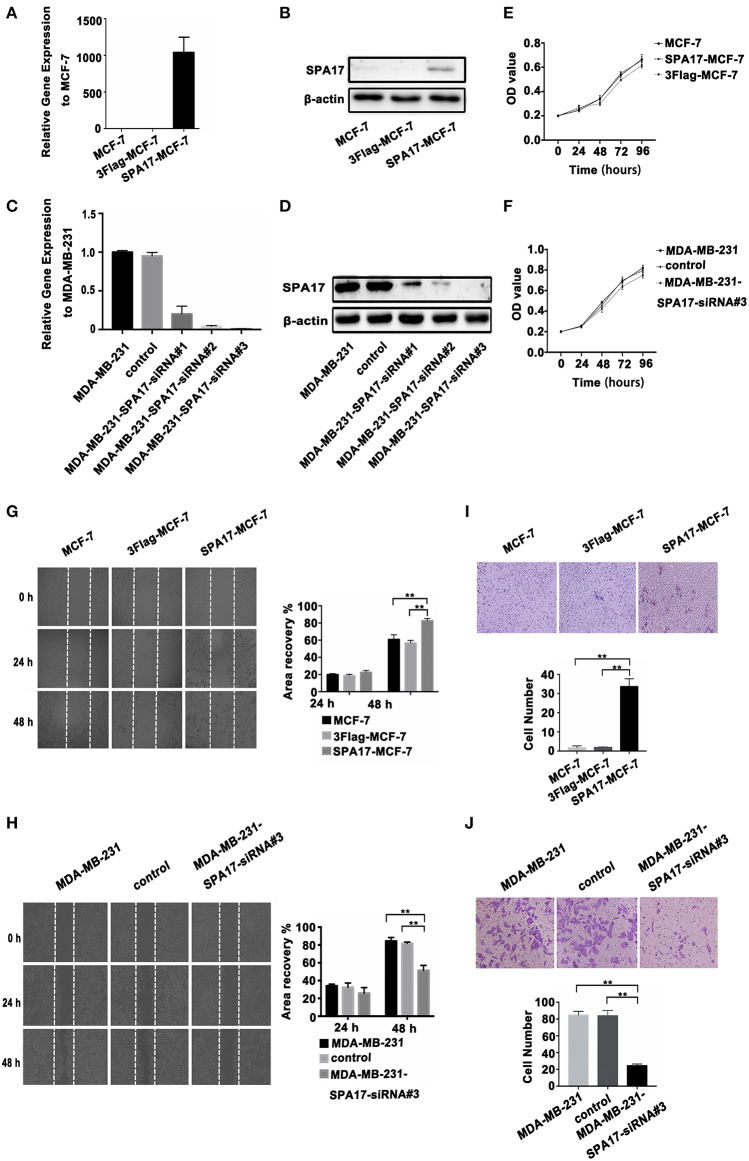
**(A,B)** The overexpression of SPA17 gene and protein in SPA17-MCF-7 cells was confirmed by Q-PCR and Western-blot. **(C,D)** The silencing of SPA17 gene in MDA-MB-231 cells was confirmed by Q-PCR and Western-blot. **(E,F)** Analyses of cell growth curves using the MTT assay. **(G)** The migration of SPA17-MCF-7, 3Flag-MCF-7, MCF-7, **(H)** MDA-MB-231, control and MDA-MB-231-SPA17-siRNA#3 cells were measured with the wound-healing assay (*n* = 3). After culturing for 24 h and 48 h, the cells were photographed at ×40; Samples were compared using one-way ANOVA, and error bars represent mean ± S.D. **(I)** The invasion of the SPA17-MCF-7, 3Flag-MCF-7, MCF-7, **(J)** MDA-MB-231, control and MDA-MB-231-SPA17-siRNA#3 cells were measured using the transwell assay (*n* = 3). After culturing for 48 h, the cells were stained with crystal violet solution and photographed at 200. Samples were compared using one-way ANOVA, and error bars represent mean ± S.D., ^*^p < 0.05, ^**^p < 0.01.

We found that the expression of SPA17 may be related to the metastasis of breast cancer as mentioned above. The lowest and the highest expression of SPA17 were found in Luminal A and triple-negative subtype breast cancer, respectively (Figure 2H). Therefore, we selected the MCF-7 and the MDA-MB-231 cell line for functional verification of SPA17 overexpression and silencing, and Q-PCR and western blot confirmed that MCF-7 breast cancer cells hardly expressed SPA17, while MDA-MB-231 breast cancer cells highly expressed SPA17. After MCF-7 cells were infected with plasmids containing either SPA17 cDNA or EGFP cDNA, the overexpression of SPA17 in SPA17-MCF-7 cells was confirmed by Q-PCR and western blot (Figures 4A,B). MCF-7 and 3Flag-MCF-7 cells were used as controls (*p* < 0.01). After MDA-MB-231 cells were infected with target siRNAs or control siRNA, the silencing of SPA17 in MDA-MB-231 cells was also confirmed by Q-PCR and western blot (Figures 4C,D). As can be seen from Figure 4D, siRNA-SPA17#3 had the best silencing result, so it was used for further analysis.

We performed the MTT assay showed that SPA17 overexpression did not increased the proliferation of MCF-7, while SPA17 depletion inhibited the proliferation rate of MDA-MB-231 breast cancer cells slightly, but the differences were not statistically significant (*p* > 0.05) (Figures 4E,F).

The wound healing assay indicated that the gap in the SPA17-MCF-7 cell monolayer was slightly faster closed over 24 h compared with those in the control groups, but the differences were not statistically significant (SPA17-MCF-7 vs. MCF-7, *p* = 0.144; SPA17-MCF-7 vs. 3Flag-MCF-7, *p* = 0.112; 3Flag-MCF-7 vs. MCF-7, *p* = 0.329; Figure 4G). However, the gap in the SPA17-MCF-7 cells was closed more rapidly over 48 h compared with those in the control groups (SPA17-MCF-7 vs. MCF-7, *p* < 0.001; SPA17-MCF-7 vs. 3Flag-MCF-7, *p* = 0.004; 3Flag-MCF-7 vs. MCF-7, *p* = 0.285; Figure 4G). The gap in the MDA-MB-231 cell monolayer was slightly faster closed over 24 h compared with those in the control groups, but the differences were not statistically significant (MDA-MB-231 vs. MDA-MB-231-siRNA, *p* = 0.099; control vs. MDA-MB-231-siRNA, *p* = 0.227; MDA-MB-231 vs. control, *p* = 0.616; Figure 4H). However, the gap in the MDA-MB-231 cells was closed more rapidly over 48 h compared with those in the control groups (MDA-MB-231 vs. MDA-MB-231-siRNA, *p* = 0.001; control vs. MDA-MB-231-siRNA, *p* = 0.006; MDA-MB-231 vs. control, *p* = 0.218; Figure 4H). These results demonstrated that SPA17 increased the migration ability of breast cancer cells.

In the Transwell assay, the number of SPA17-MCF-7 cells that migrated through the polycarbonate membrane was significantly higher than that of MCF-7 cells at 48 h (SPA17-MCF-7 vs. MCF-7, *p* = 0.007; SPA17-MCF-7 vs. 3Flag-MCF-7, *p* = 0.009; 3Flag-MCF-7 vs. MCF-7, *p* = 0.478; Figure 4I). However, the number of MDA-MB-231 cells that migrated through the polycarbonate membrane was significantly higher than that of MDA-MB-231-siRNA cells at 48 h (MDA-MB-231 vs. MDA-MB-231-siRNA, *p* < 0.001; control vs. MDA-MB-231-siRNA, *p* < 0.001; MDA-MB-231 vs. control, *p* = 0.840; Figure 4J).These results demonstrated that SPA17 increased the invasion ability of breast cancer cells.

## Discussion

In this study, we systematically studied the expression of SPA17 in breast cancer tissues and its function in the development of breast cancer. We found that SPA17 was expressed at the mRNA and protein levels only in breast cancer specimens, especially in triple-negative (60%) or HER2-positive (45%) subtypes, and not in normal breast tissues. With further analysis, we also observed that the expression of SPA17 was higher in patients with lymph node metastasis (55.6%), Cox regression analysis showed SPA17 was an independent factor associated with DFS and OS in breast cancer patients. *In vitro* mechanism study showed that high expression of SPA17 significantly increased the migration and invasion of MCF-7 breast cancer cells, on the contrary, low expression of SPA17 significantly inhibited the migration and invasion of MDA-MB-231 breast cancer cells, which indicates that SPA17 expression may be the cause of poor prognosis in breast cancer patients.

In terms of SPA17 protein positive expression rate in breast cancer tissues, our rate was relatively high, which up to 27%. A previous study conducted by Gjerstorff and Ditzel reported the overall expression of SPA17 to be 12% in breast cancer patients they reviewed (20), which is lower than what we observed in our study. Besides, the expression of SPA17 was also related to molecular subtyping, it expressed more in triple-negative (60%) or HER2 positive (45%) breast cancers. Based on these, we speculated that the reason why SPA17 expression rate in our study was higher might be that we screened patients to balance the number of patients in each molecular subtyping. Whatever, these studies have confirmed that SPA17 expressed in breast cancer, and its expression had a relationship with molecular subtyping. The results of this study were consistent with the results of Oncomine dataset.

On the other hand, the presence of SPA17 has been linked to metastases in previous studies. Li et al. reported that overexpression of SPA17 in the ovarian cancer cell line HO-8910 increased cellular metastasis (22). Besides, some other CTAs have been reported to promote breast cancer metastasis by various mechanisms (17–20). Previously, whether the expression of SPA17 is related to breast cancer prognosis has not yet been reported. In this study, we analyzed the status of SPA17 expression and lymph node metastasis in patients. We observed that the positive expression rate of SPA17 was higher in patients with lymph node metastasis, indicating that SPA17 expression likely promoted lymph node metastasis in breast cancer. Further survival analysis also suggested that the expression of SPA17 indicated a poor prognosis.

Based on the above analysis, it is reasonable that the expression of SPA17 could affect the migration and invasion of breast cancer cells. Considering the lowest expression and the highest expression of SPA17 in Luminal A breast cancer and triple-negative breast cancer, respectively, we selected MCF-7 and MDA-MB-231 cells to verify the role of SPA17 in regulating the biological function of breast cancer cells. Previously, it was found that MCF-7 cells did not express SPA17, and their migration and invasion abilities were relatively weak compared to those of the other three subtypes of breast cancer cells. However, after SPA17 transfection, the migration and invasion ability of MCF-7 cells were significantly enhanced. Comparetively, MDA-MB-231 cells highly expressed SPA17, but after transfecting with SPA17-siRNA, the migration and invasion ability of MDA-MB-231 cells were significantly inhibited. These results strongly supported the necessity to explore the mechanism of SPA17. Though Lea et al. found that SPA17 can combine AKAP3 binding sites on protein kinase alpha adjust II subunit, and interrupt the main interaction between AKAP3 and PKA, thus leading to malpositioning of PKA and thus interfering with its regulatory role in specific subcellular locations and key signal transduction pathways (23, 24). However, whether localization of SPA17-AKAP to a particular subcellular site will be enough for tumor development remains to be further explored.

All of our results indicated that SPA17 plays a vital role in the development, migration, and invasion of breast cancer and that it can be used as an important diagnostic and prognostic indicator in clinical practice. Combined with relevant immunological research and mechanism exploration, SPA17 may be a new therapeutic target to improve the prognosis of breast cancer, especially the triple-negative and HER2-positive subtypes, in the future.

## Conclusion

Our findings suggest a role for SPA17 in tumor metastasis and aggressiveness. Whether SPA17 can be used as a prognostic marker for breast cancer will require a study of a larger sample size.

## Data Availability

No datasets were generated or analyzed for this study.

## Author Contributions

YZ, JQ, YW, PL, and QL proposed the study concept and design. YZ, JQ, and YW performed the research and acquired the data. JQ, YZ, and PL analyzed and interpreted the data. YZ and JQ wrote the first draft and provided a critical revision of the manuscript with important intellectual content. QL and ZD were the study supervisor.

### Conflict of Interest Statement

The authors declare that the research was conducted in the absence of any commercial or financial relationships that could be construed as a potential conflict of interest.
