# Corrigendum: Sperm Protein Antigen 17 Expression Correlates With Lymph Node Metastasis and Worse Overall Survival in Patients With Breast Cancer

**DOI:** 10.3389/fonc.2020.01003

**Published:** 2020-06-24

**Authors:** Yu-ting Zhou, Juan-juan Qiu, Yao Wang, Peng-cheng Liu, Qing Lv, Zheng-gui Du

**Affiliations:** West China Hospital, Sichuan University, Chengdu, China

**Keywords:** sperm protein antigen 17, cancer-testis antigens, migration, invasion, breast cancer

In the published article, Zheng-gui Du was not included as a corresponding author.

The authors apologize for this error and state that this does not change the scientific conclusions of the article in any way. The original article has been updated.

